# MicroRNA-34a Promotes Hepatic Lipid Accumulation Through RXRα Suppression and Is Reversed by 9-*cis*-Retinoic Acid in Steatotic Hepatocytes

**DOI:** 10.3390/ijms27156609

**Published:** 2026-07-24

**Authors:** Jai-Sing Yang, Hong-Yi Chiu, Syun-Rong Jhan, Yao-An Liu, Chao-Jung Chen, Shih-Chang Tsai

**Affiliations:** 1 Department of Medical Research, China Medical University Hospital, China Medical University, Taichung 404327, Taiwan; 2 Department of Pharmacy, Hualien Tzu Chi Hospital, Buddhist Tzu Chi Medical Foundation, Hualien 970473, Taiwan; 3 Graduate Institute of Clinical Pharmacy, College of Medicine, Tzu Chi University, Hualien 970473, Taiwan; 4 Department of Pharmacy, College of Medicine, Tzu Chi University, Hualien 970473, Taiwan; 5 Department of Biological Science and Technology, China Medical University, Taichung 406040, Taiwanalan92926@gmail.com (Y.-A.L.); 6 Graduate Institute of Integrated Medicine, China Medical University, Taichung 404327, Taiwan

**Keywords:** metabolic dysfunction-associated steatotic liver disease (MASLD), microRNA-34a, retinoid X receptor alpha (RXRα), hepatic steatosis, lipid homeostasis, nuclear receptor signaling, therapeutic target

## Abstract

Metabolic dysfunction-associated steatotic liver disease (MASLD) is the most prevalent chronic liver disease worldwide and is characterized by excessive hepatic lipid accumulation and metabolic dysfunction. Although microRNA-34a (miR-34a) has been implicated in hepatic lipid metabolism, the molecular mechanisms underlying its contribution to MASLD remain incompletely understood. Here, we investigated the role of miR-34a in FFA-induced hepatic steatosis using HepG2 cells and explored RXR-associated signaling as a potential therapeutic strategy. RT-qPCR quantified miR-34a expression; direct target interactions were validated using dual-luciferase reporter assays; proteomic alterations were characterized by iTRAQ-based proteomics followed by Ingenuity Pathway Analysis (IPA); and transcriptomic responses to 9-*cis*-retinoic acid (9-*cis*-RA) were analyzed by RNA sequencing. FFA treatment significantly increased miR-34a expression, and dual-luciferase assays confirmed that miR-34a directly targets the 3′-UTRs of RXRα, PPARα, and SIRT1. Integrated proteomic and transcriptomic analyses consistently identified LXR/RXR signaling as one of the principal pathways associated with miR-34a dysregulation and 9-*cis*-RA treatment. Pharmacological activation of RXR-associated signaling with the pan-RXR agonist 9-*cis*-RA attenuated intracellular lipid accumulation and reduced the expression of key regulators of lipogenesis and fatty acid uptake, including FASN, SCD1, FABP4, and CD36. Collectively, these findings support the miR-34a–RXRα axis as one regulatory component within a broader nuclear receptor network associated with hepatic lipid homeostasis and support further investigation of RXR-associated signaling as a potential therapeutic strategy for MASLD. Nevertheless, confirmation of receptor-specific mechanisms and validation in more physiologically relevant experimental models will be required.

## 1. Introduction

In 2023, an international multi-society Delphi consensus introduced the metabolic dysfunction-associated steatotic liver disease (MASLD) nomenclature, replacing the previous terms nonalcoholic fatty liver disease (NAFLD) and metabolic dysfunction-associated fatty liver disease (MAFLD), to provide a more inclusive and clinically relevant classification based on the presence of hepatic steatosis and cardiometabolic risk factors [1,2]. MASLD is now recognized as the most prevalent chronic liver disease worldwide, affecting approximately 25–30% of the global population and closely associated with obesity, insulin resistance, type 2 diabetes, and metabolic syndrome [1,2]. MASLD encompasses a pathological spectrum ranging from simple hepatic steatosis to metabolic dysfunction-associated steatohepatitis (MASH), which can progress to fibrosis, cirrhosis, and hepatocellular carcinoma [3,4]. Despite recent therapeutic advances, effective pharmacological therapies remain limited, and elucidating the molecular mechanisms underlying MASLD remains an important research priority.

MASLD pathogenesis is multifactorial and involves dysregulated lipid metabolism, chronic inflammation, oxidative stress, and insulin resistance. Hepatic lipid accumulation results from an imbalance between lipid acquisition (fatty acid uptake and de novo lipogenesis) and lipid disposal (fatty acid β-oxidation and very-low-density lipoprotein secretion) [3]. In addition to metabolic disturbances, epigenetic regulators, particularly microRNAs (miRNAs), have emerged as critical modulators of hepatic lipid homeostasis [5,6]. miRNAs are small non-coding RNAs that regulate gene expression post-transcriptionally through complementary binding to target mRNAs, thereby influencing diverse biological processes [5]. Aberrant miRNA expression has been implicated in the dysregulation of lipid metabolism, insulin signaling, inflammation, and fibrosis during MASLD progression [7,8].

Among these miRNAs, miR-34a is one of the most consistently upregulated in MASLD and related metabolic disorders [8,9]. As a downstream effector of p53 signaling, it regulates multiple cellular processes, including apoptosis, mitochondrial function, lipid metabolism, and metabolic stress response [10,11]. Accumulating evidence indicates that metabolic stress, lipotoxicity, and inflammatory stimuli induce miR-34a expression [12,13], and elevated hepatic miR-34a levels have consistently been observed in both patients with MASLD and experimental models, supporting its involvement in disease progression [9,14]; nevertheless, the downstream molecular mechanisms through which it promotes hepatic lipid accumulation remain incompletely characterized.

Retinoid X receptor alpha (RXRα) is a ligand-activated nuclear receptor that functions as an essential heterodimeric partner for several metabolic regulators, including peroxisome proliferator-activated receptors (PPARs), liver X receptors (LXRs), farnesoid X receptor (FXR), and retinoic acid receptors (RARs) [15,16,17]. Through these heterodimeric interactions, RXRα coordinates transcriptional programs governing lipid metabolism, fatty acid oxidation, cholesterol transport, and bile acid homeostasis [10,18]. Impaired RXRα signaling has been associated with hepatic steatosis, metabolic dysfunction, and progression of fatty liver disease, supporting an important role in maintaining hepatic metabolic homeostasis [18,19,20].

Recent studies have identified RXRα as a direct target of miR-34a, establishing a mechanistic connection between miRNA-mediated regulation and nuclear receptor signaling [6,20,21]. However, although this interaction has been described in hepatic stellate cells during liver fibrosis, its functional significance in hepatocyte steatosis and its integration into broader nuclear receptor signaling networks remain poorly understood [21]. Therefore, the present study aimed to define the contribution of the miR-34a–RXRα axis to hepatic lipid accumulation through integrated proteomic and transcriptomic analyses. Suppression of RXRα by miR-34a may therefore disrupt multiple downstream metabolic pathways associated with hepatic lipid homeostasis. Accordingly, pharmacological activation of RXR-associated signaling has emerged as a potential therapeutic strategy for MASLD. Among currently available RXR agonists, 9-*cis*-retinoic acid (9-*cis*-RA) is the endogenous high-affinity ligand for RXRs and activates multiple RXR-associated transcriptional programs, including LXR/RXR signaling, thereby improving lipid metabolism and attenuating hepatic steatosis [22,23,24,25]. Because 9-*cis*-RA activates multiple RXR isoforms and also engages RAR signaling, it is regarded throughout this study as a pan-RXR agonist rather than a selective RXRα activator. However, whether pharmacological activation of RXR-associated signaling can attenuate the metabolic dysregulation associated with miR-34a remains unclear.

In the present study, we investigated the regulatory relationship between miR-34a and RXRα in MASLD and evaluated the therapeutic potential of RXR-associated signaling activated by 9-*cis*-RA. We hypothesized that miR-34a promotes hepatic lipid accumulation, at least in part, by suppressing RXRα, and that pharmacological activation of RXR-associated signaling may attenuate these metabolic alterations. Through integrated proteomic, transcriptomic, molecular, functional, and bioinformatic analyses, we aimed to define the miR-34a–RXRα regulatory network, provide mechanistic insight into its role in hepatic lipid homeostasis, and evaluate whether pharmacological activation of RXR-associated signaling attenuates hepatic lipid accumulation.

## 2. Results

### 2.1. miR-34a Is Upregulated in FFA-Treated Steatotic Hepatocytes and Directly Targets PPARα and SIRT1

**Figure 1:**
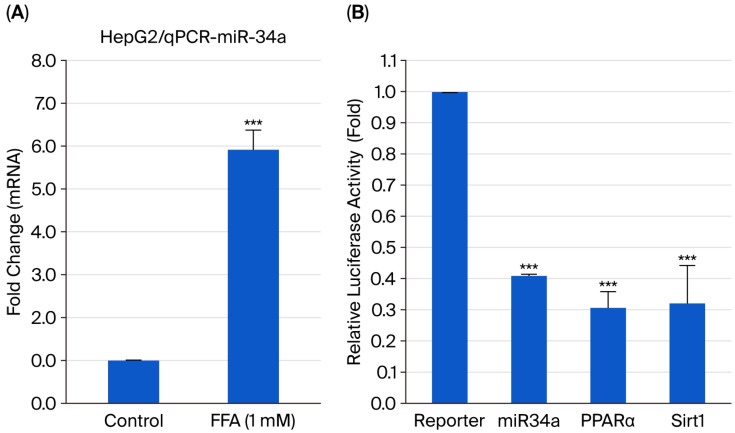
miR-34a is upregulated by free fatty acid treatment and directly targets key regulators of lipid metabolism in HepG2 cells. (**A**) RT-qPCR analysis showing significantly increased miR-34a expression in HepG2 cells treated with 1 mM free fatty acids (FFAs; oleic acid: palmitic acid = 2:1, 0.67 mM OA and 0.33 mM PA) for 24 h, compared with vehicle-treated controls. (**B**) Dual-luciferase reporter assays showing that transfection of an miR-34a mimic significantly reduced the luciferase activity of reporter constructs containing the 3′-untranslated regions (3′-UTRs) of PPARα and SIRT1 in HepG2 cells, consistent with direct regulation by miR-34a. Data are presented as the mean ± SD of three independent experiments. *** *p* < 0.001 vs. the corresponding control.

To determine whether miR-34a is altered during FFA-induced steatosis, HepG2 cells were treated with free fatty acids (FFAs; oleic acid and palmitic acid at a 2:1 ratio, final concentration 1 mM) for 24 h. RT-qPCR demonstrated that miR-34a expression was significantly increased following FFA treatment compared with vehicle-treated controls (*p* < 0.001), indicating increased miR-34a expression under steatotic conditions (Figure 1A).

Bioinformatic prediction identified peroxisome proliferator-activated receptor alpha (PPARα) and sirtuin 1 (SIRT1) as putative targets of miR-34a [26,27]. To validate these predicted interactions, dual-luciferase reporter assays were performed in HepG2 cells. Overexpression of miR-34a significantly reduced the luciferase activity of reporter constructs containing the 3′-untranslated regions (3′-UTRs) of both PPARα and SIRT1 (both *p* < 0.001), confirming direct targeting by miR-34a (Figure 1B) [9,26].

These results demonstrate that miR-34a expression is increased in FFA-treated HepG2 cells and directly targets PPARα and SIRT1.

### 2.2. iTRAQ-Based Proteomic Profiling Identifies Widespread Alterations in Lipid Metabolism and RXR-Associated Nuclear Receptor Signaling Following miR-34a Overexpression

Quantitative iTRAQ-based proteomic profiling was performed to investigate the global proteomic alterations induced by miR-34a overexpression in HepG2 cells. A total of 6907 proteins were identified, of which 6623 were successfully quantified. Because the proteomic analysis was performed in the absence of free fatty acid (FFA) loading, the resulting dataset reflects the intrinsic molecular consequences of miR-34a overexpression rather than secondary changes associated with steatosis, thereby complementing the functional observations obtained from the FFA-induced HepG2 model. Given that the proteomic analysis included two biological replicates per group and differentially expressed proteins were identified using fold-change thresholds alone, the dataset should be regarded as an exploratory, hypothesis-generating resource. Key findings from this proteomic screen, including the direct regulation of RXRα by miR-34a, were subsequently validated by an independent dual-luciferase reporter assay, as described in Section 2.3.

**Figure 2:**
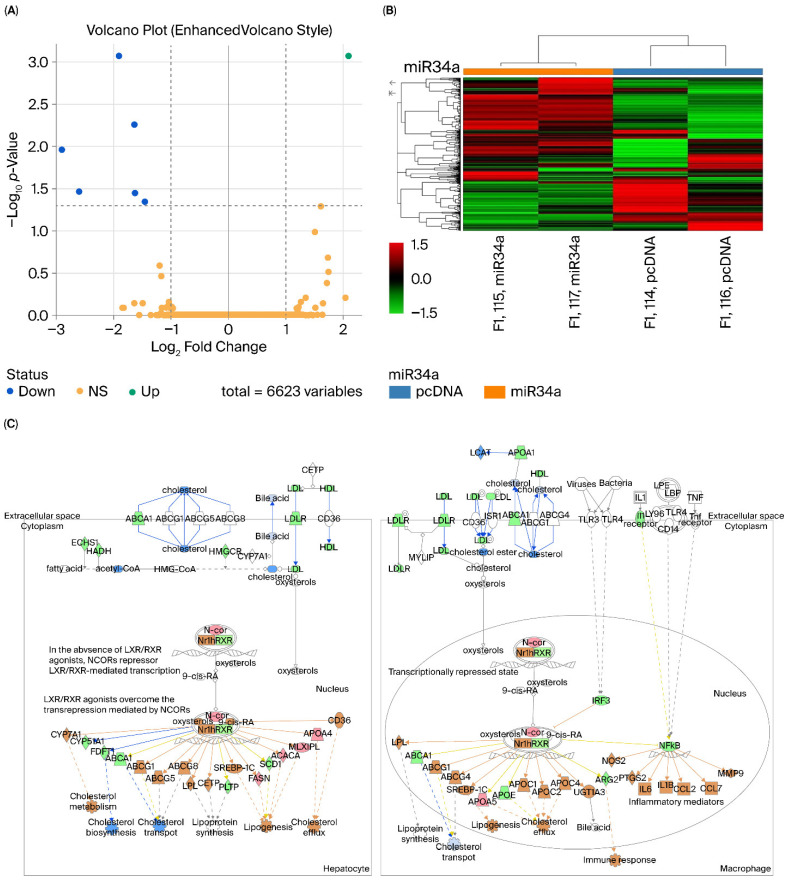
iTRAQ-based proteomic profiling reveals miR-34a-associated alterations in lipid metabolism and nuclear receptor signaling pathways. (**A**) Volcano plot showing differentially expressed proteins (DEPs) in miR-34a-overexpressing HepG2 cells compared with pcDNA-transfected controls (6623 proteins identified and quantified). Red, green, and gray dots represent proteins with increased abundance (abundance ratio ≥ 1.2), decreased abundance (abundance ratio ≤ 0.83), and no substantial change, respectively. (**B**) Unsupervised hierarchical clustering heatmap of DEPs showing distinct proteomic profiles between miR-34a-overexpressing cells (orange bar, *n* = 2) and pcDNA-transfected controls (blue bar, *n* = 2). Relative protein abundance is displayed as row-wise z-scores ranging from −1.5 (green) to 1.5 (red). (**C**) Ingenuity Pathway Analysis (IPA) of DEPs showing enrichment of canonical pathways associated with LXR/RXR activation, cholesterol biosynthesis and transport, lipoprotein metabolism, fatty acid metabolism, cholesterol homeostasis, and inflammatory signaling. LXR/RXR activation was one of the most prominently enriched canonical pathways identified by IPA.

Using abundance ratio thresholds of ≥1.2 and ≤0.83, miR-34a overexpression led to the upregulation of 2508 proteins and the downregulation of 1610 proteins, indicating broad proteomic remodeling (Figure 2A). Unsupervised hierarchical clustering readily distinguished miR-34a-overexpressing cells from control cells, revealing a distinct proteomic signature associated with miR-34a overexpression (Figure 2B).

To further characterize the biological pathways affected by miR-34a, differentially expressed proteins were analyzed using Ingenuity Pathway Analysis (IPA). Pathway enrichment analysis identified significant enrichment of pathways associated with lipid metabolism and inflammatory regulation, including LXR/RXR activation, cholesterol biosynthesis and transport, lipoprotein metabolism, fatty acid metabolism, and cholesterol homeostasis (Figure 2C). Among these, LXR/RXR activation was one of the most significantly enriched canonical pathways. IPA also identified multiple RXR-associated regulatory networks associated with lipid metabolism and inflammatory signaling. These findings are consistent with altered RXR-associated signaling following miR-34a overexpression.

In addition, FOXO3 and several FOXO-associated signaling components were detected in the proteomic dataset. FOXO3 abundance was reduced following miR-34a overexpression, consistent with previous reports identifying FOXO3 as a direct target of miR-34a associated with oxidative stress responses and metabolic regulation (Supplementary Table S1). These findings are consistent with miR-34a coordinating the regulation of multiple metabolic transcriptional regulators rather than modulating a single downstream effector.

Collectively, the proteomic analysis demonstrated widespread alterations in metabolic and inflammatory pathways following miR-34a overexpression. The enrichment of RXR-associated nuclear receptor signaling, together with the independent validation of RXRα as a direct miR-34a target by dual-luciferase reporter assays, is consistent with the involvement of RXR-associated signaling in miR-34a-mediated metabolic regulation. The complete proteomic dataset is provided in Supplementary Table S1.

### 2.3. miR-34a Directly Targets the RXRα 3′-UTR and Suppresses Reporter Activity

**Figure 3:**
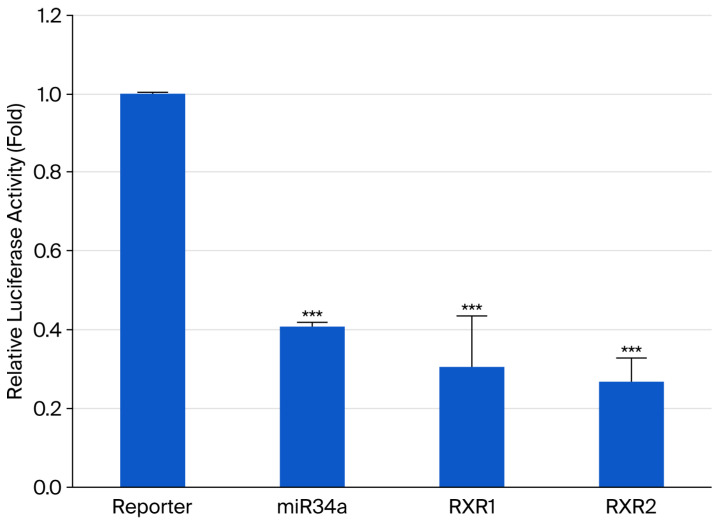
miR-34a directly targets the RXRα 3′-UTR and suppresses reporter activity. Dual-luciferase reporter assays show that transfection with an miR-34a mimic significantly reduced luciferase activity in reporter constructs containing the wild-type RXRα 3′-UTR (RXR1 and RXR2 binding sites). Data are presented as mean ± SD from three independent experiments. *** *p* < 0.001 versus the corresponding control group.

Based on the enrichment of LXR/RXR signaling identified by the integrative omics analyses, RXRα was selected for experimental validation as a putative downstream target of miR-34a. Bioinformatic analysis identified two conserved putative miR-34a-binding sites (RXR1 and RXR2) within the 3′-untranslated region (3′-UTR) of the RXRα mRNA (Figure 3) [21]. To validate these predicted interactions, dual-luciferase reporter assays were performed using wild-type RXRα 3′-UTR reporter constructs. Overexpression of miR-34a significantly reduced the luciferase activity of both RXR1 and RXR2 reporter constructs compared with the negative control (both *p* < 0.001). These findings demonstrate that miR-34a directly targets the RXRα 3′-UTR, thereby confirming RXRα as a direct downstream target of miR-34a.

Consistent with the reporter assay results, stable miR-34a overexpression was associated with reduced endogenous RXRα protein expression and decreased SIRT1 and PPARα protein levels (Supplementary Figure S1). These findings further support direct regulation of RXRα by miR-34a at both the reporter and endogenous protein levels.

### 2.4. Activation of RXR-Associated Signaling by 9-cis-Retinoic Acid Attenuates FFA-Induced Lipid Accumulation in Hepatocytes

**Figure 4:**
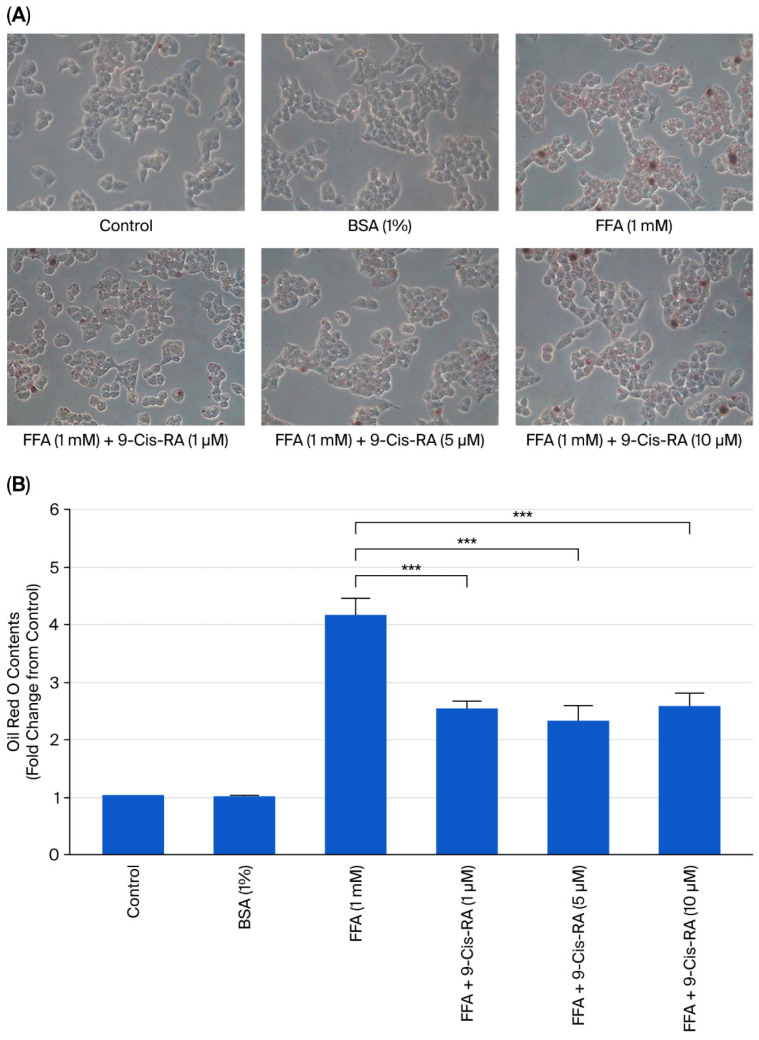

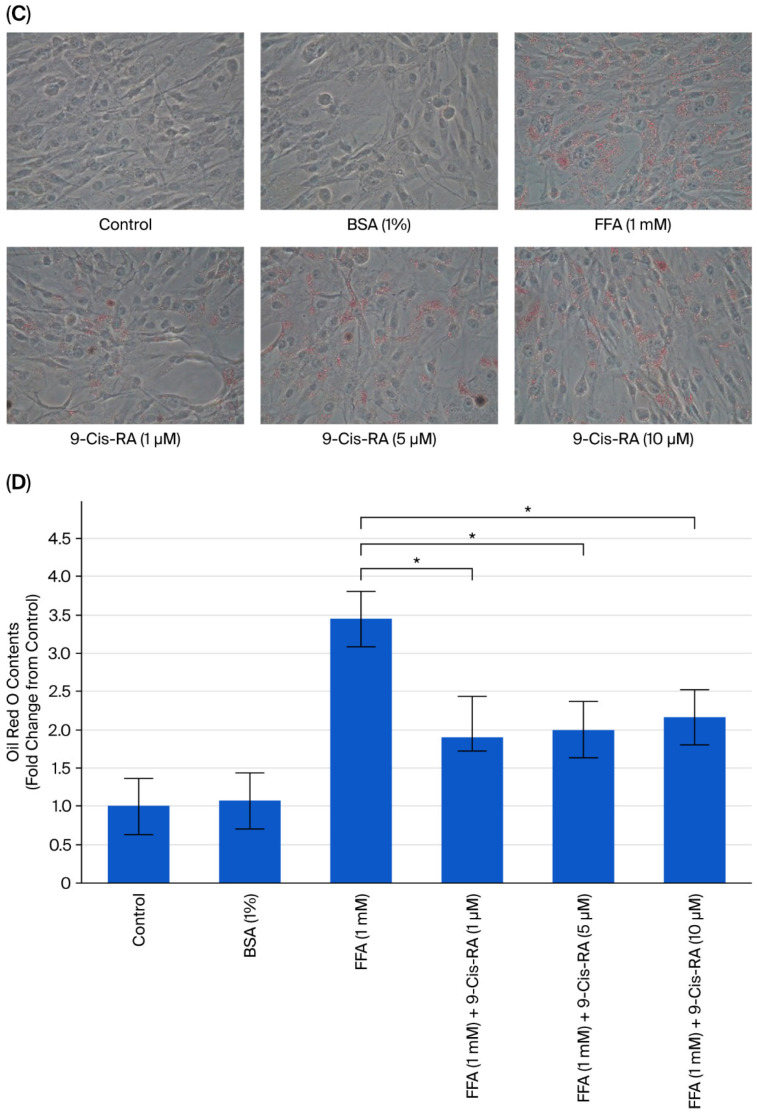
9-*cis*-Retinoic acid attenuates FFA-induced lipid accumulation in HepG2 cells and primary human hepatocytes. (**A**) Representative Oil Red O staining images of HepG2 cells treated with vehicle control, 1% BSA, free fatty acids (FFAs; 1 mM; oleic acid: palmitic acid = 2:1), or FFA in combination with 9-*cis*-retinoic acid (1, 5, or 10 μM) for 24 h. FFA treatment markedly increased intracellular lipid droplet accumulation, whereas co-treatment with 9-*cis*-retinoic acid progressively reduced lipid deposition. Representative images were acquired at 200× magnification. (**B**) Quantitative analysis of Oil Red O staining by spectrophotometric measurement at 510 nm after normalization to total protein content. FFA treatment significantly increased intracellular lipid accumulation, whereas 9-*cis*-retinoic acid significantly reduced lipid accumulation compared with the FFA-treated group. Data are presented as the mean ± SD from three independent experiments. *** *p* < 0.001 vs. the FFA-treated group. (**C**) Representative microscopic images of human hepatocytes (HH) corresponding to the experimental groups shown in (**A**), illustrating the reduction in intracellular lipid droplet accumulation following treatment with 9-*cis*-retinoic acid. (**D**) Quantitative analysis of Oil Red O staining by spectrophotometric measurement at 510 nm after normalization to total protein content. FFA treatment increased lipid accumulation by approximately 3.5-fold, whereas co-treatment with 9-*cis*-retinoic acid reduced lipid accumulation to approximately 2.0–2.2-fold of the control level. Data are presented as the mean ± SD from three independent experiments. * *p* < 0.05 vs. the FFA-treated group.

To investigate whether pharmacological activation of RXR-associated signaling attenuates lipid accumulation under steatotic conditions, FFA-loaded HepG2 cells were treated with increasing concentrations of 9-*cis*-retinoic acid (9-*cis*-RA), a pan-RXR agonist that also activates retinoic acid receptors (RARs). FFA loading markedly increased intracellular lipid accumulation, as demonstrated by abundant Oil Red O-positive lipid droplets (Figure 4A). Co-treatment with 9-*cis*-RA produced a concentration-dependent reduction in lipid droplet accumulation. Quantitative analysis of Oil Red O staining confirmed that 9-*cis*-RA significantly reduced intracellular lipid content compared with the FFA-treated group (*p* < 0.001), with the greatest reduction observed at 10 μM (Figure 4B).

Comparable lipid-lowering effects were observed in primary human hepatocytes (HH). Treatment with 9-*cis*-RA reduced FFA-induced lipid droplet accumulation and significantly decreased intracellular lipid content compared with FFA treatment alone (Figure 4C,D). Together, these findings indicate that the lipid-lowering effect of 9-*cis*-RA is reproducible in both HepG2 cells and primary human hepatocytes.

Collectively, these results demonstrate that 9-*cis*-RA attenuates FFA-induced lipid accumulation in steatotic hepatocytes.

### 2.5. Transcriptomic Analysis Reveals Extensive Gene Expression Remodeling Following 9-cis-Retinoic Acid Treatment in Steatotic Hepatocytes

**Figure 5:**
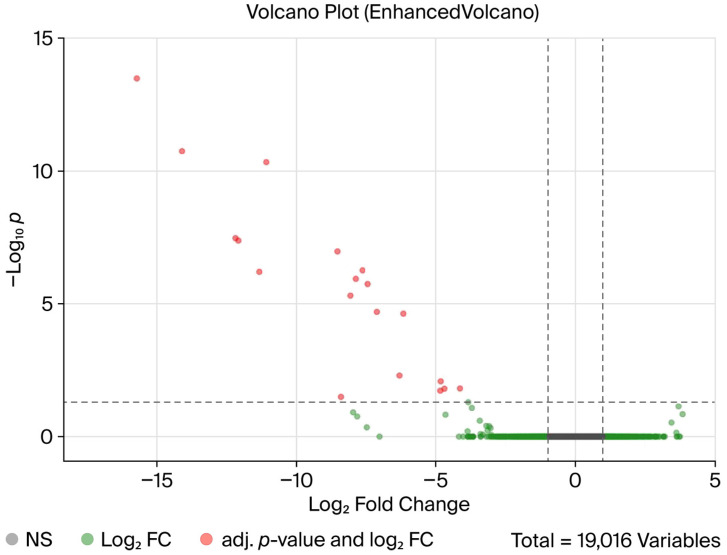
Transcriptomic alterations induced by 9-*cis*-retinoic acid in FFA-treated HepG2 cells. Volcano plot showing differentially expressed genes (DEGs) identified by RNA sequencing in HepG2 cells treated with FFA plus 10 μM 9-*cis*-retinoic acid compared with FFA-treated cells. RNA sequencing detected 19,016 transcripts across all samples, and differential expression analysis was performed following transcript-to-gene summarization and quality filtering, as described in the text. Red, green, and gray dots indicate genes with adjusted *p* < 0.05 and |log_2_ fold change| > 1, genes with |log_2_ fold change| > 1 but adjusted *p* ≥ 0.05, and genes without significant differential expression, respectively.

To characterize the global transcriptional responses to 9-*cis*-retinoic acid (9-*cis*-RA), RNA sequencing was performed in FFA-treated HepG2 cells with or without 10 μM 9-*cis*-RA. Transcriptomic profiling revealed widespread changes in gene expression following 9-*cis*-RA treatment, demonstrating extensive alterations in steatotic hepatocytes. Differential expression analysis (adjusted *p* < 0.05 and |log_2_ fold change| > 1) identified a distinct set of significantly regulated genes, as illustrated by the volcano plot (Figure 5). Compared with the FFA-treated group, co-treatment with 9-*cis*-RA upregulated 551 genes and downregulated 826 genes, indicating substantial transcriptional changes.

RNA sequencing detected a total of 19,016 transcripts across all samples. Following transcript-to-gene summarization and quality filtering, 18,836 genes with valid expression values were retained for differential expression analysis between the FFA and FFA + 9-*cis*-RA groups (Supplementary Table S2). The complete RNA-seq expression matrix, comprising 60,623 Ensembl gene entries across all treatment groups and pairwise comparisons, is provided in Supplementary Table S2.

Collectively, these findings demonstrate that treatment with 9-*cis*-RA is associated with widespread transcriptomic alterations in FFA-treated HepG2 cells.

### 2.6. IPA Reveals Coordinated Regulation of Lipid Metabolism and Inflammatory Signaling Following 9-cis-Retinoic Acid Treatment

**Figure 6:**
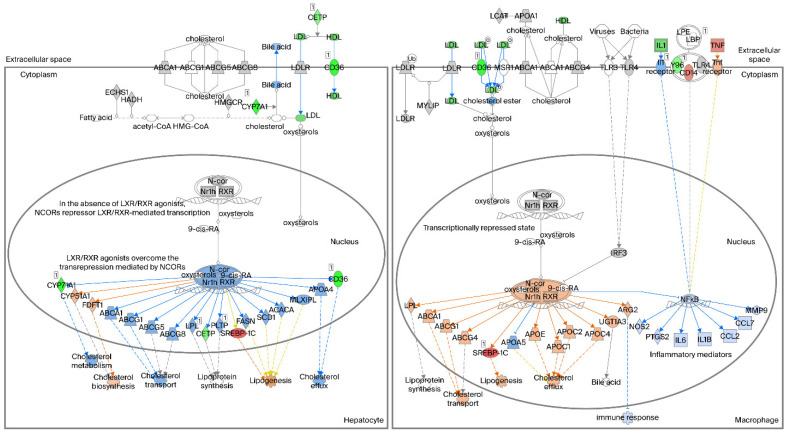
Ingenuity Pathway Analysis (IPA) illustrates inferred LXR/RXR-associated transcriptional networks following 9-*cis*-retinoic acid treatment. IPA canonical pathway diagrams generated from RNA-seq data of HepG2 cells treated with FFA plus 10 μM 9-*cis*-retinoic acid are shown. The predicted regulatory networks for hepatocytes (**left**) and macrophages (**right**) highlight LXR/RXR-associated sig-naling. In the hepatocyte network, genes associated with cholesterol transport, cholesterol homeostasis, and bile acid metabolism, including ABCA1, ABCG1, ABCG5/8, CYP7A1, and multiple apolipoproteins, are shown together with lipid metabolism-related genes, including FASN, SCD1, and SREBP-1C. The macrophage network includes inflammatory mediators in NF-κB-associated signaling pathways, such as IL6, IL1B, CCL2, and MMP9. Node colors indicate the direction of expression or predicted activity: red/orange represents increased expression or predicted activation, whereas green/blue represents decreased expression or predicted inhibition, with color intensity reflecting the magnitude of change or prediction. Uncolored (white/gray) nodes represent molecules for which no expression change was identified in the dataset or whose activity was inferred from the network. Different node shapes represent different molecular or functional classes, as defined by IPA. Solid and dashed lines indicate direct and indirect relationships, respectively, while arrow colors reflect the predicted direction or consistency of the regulatory relationship based on IPA. Numbers displayed adjacent to or above nodes indicate the number of molecules represented or grouped within the corresponding node, as defined by the IPA pathway display.

Ingenuity Pathway Analysis (IPA) was performed to identify biological pathways associated with the transcriptomic changes induced by 9-*cis*-retinoic acid (9-*cis*-RA). Among the significantly enriched IPA canonical pathways, LXR/RXR activation was one of the most significantly enriched following 9-*cis*-RA treatment (Figure 6). Within hepatocyte-associated networks, IPA predicted a transcriptional program characterized by increased expression of genes associated with cholesterol efflux, lipoprotein metabolism, and bile acid synthesis, including ABCA1, ABCG1, ABCG5/8, CYP7A1, and multiple apolipoproteins. In contrast, genes associated with fatty acid synthesis and lipid accumulation, including FASN, SCD1, and SREBP-1C, were predicted to be negatively regulated [28,29,30,31].

In addition, IPA predicted reduced activity of NF-κB-associated inflammatory signaling within macrophage-related networks. Several pro-inflammatory mediators, including IL6, IL1B, CCL2, and MMP9, were likewise predicted to be downregulated following 9-*cis*-RA treatment [32]. Collectively, IPA identified coordinated transcriptional changes involving lipid metabolism and inflammatory signaling following 9-*cis*-RA treatment.

### 2.7. 9-cis-Retinoic Acid Dose-Dependently Reduces the Expression of Lipogenic and Fatty Acid Uptake Proteins

**Figure 7:**
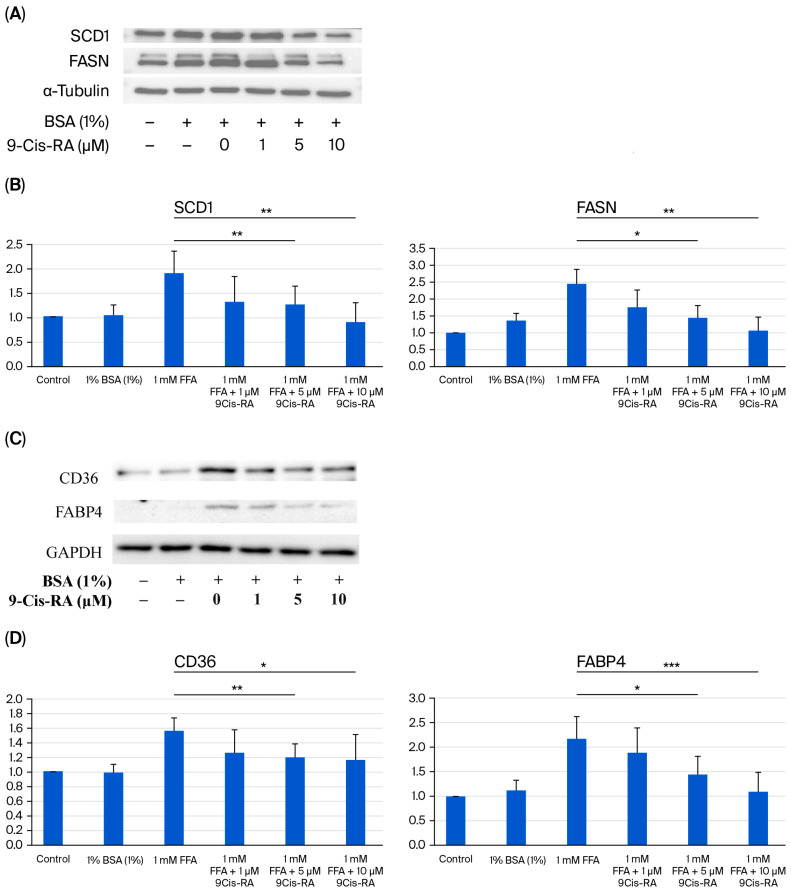
9-*cis*-Retinoic acid attenuates FFA-induced expression of proteins associated with hepatic lipid metabolism in HepG2 cells. (**A**,**C**) Representative Western blot images of SCD1, FASN, CD36, and FABP4 in HepG2 cells treated with vehicle control, 1% BSA, free fatty acids (FFAs; 1 mM), or FFAs in combination with 9-*cis*-retinoic acid (0, 1, 5, or 10 μM) for 24 h. α-Tubulin (**A**) and GAPDH (**C**) served as loading controls. (**B**,**D**) Quantitative densitometric analysis of Western blot results. FFA treatment markedly increased the protein expression of the lipogenic enzymes SCD1 and FASN, as well as the fatty acid uptake-associated proteins CD36 and FABP4. Co-treatment with 9-*cis*-retinoic acid attenuated these FFA-induced increases in a concentration-dependent manner. Data are presented as the mean ± SD from at least three independent experiments. * *p* < 0.05, ** *p* < 0.01, *** *p* < 0.001 versus the FFA-treated group.

To validate the transcriptomic findings at the protein level, Western blot analyses were performed to examine key regulators of de novo lipogenesis and fatty acid uptake in FFA-treated HepG2 cells following treatment with increasing concentrations of 9-*cis*-retinoic acid (9-*cis*-RA). FFA treatment markedly increased the protein expression of stearoyl-CoA desaturase 1 (SCD1), fatty acid synthase (FASN), fatty acid-binding protein 4 (FABP4), and cluster of differentiation 36 (CD36) (Figure 7A,C). Co-treatment with 9-*cis*-RA attenuated these FFA-induced increases in a concentration-dependent manner, with α-tubulin serving as the loading control.

Quantitative densitometric analyses confirmed significant reductions in the expression of SCD1, FASN, FABP4, and CD36 following 9-*cis*-RA treatment (Figure 7B,D). The greatest reduction in protein expression was observed at 10 μM 9-*cis*-RA. These findings are consistent with reduced expression of key proteins associated with de novo lipogenesis and fatty acid uptake following 9-*cis*-RA treatment.

### 2.8. Proposed Working Model of the miR-34a–RXRα Regulatory Axis in MASLD

**Figure 8:**
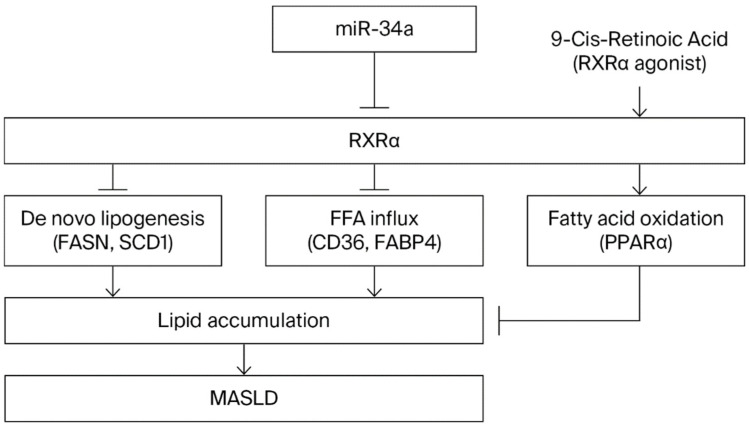
Proposed model of the miR-34a–RXRα regulatory axis in MASLD and its modulation by 9-*cis*-retinoic acid: schematic illustration summarizing the proposed findings of this study. Under steatotic conditions, FFA-induced upregulation of miR-34a is proposed to suppress RXRα expression, thereby promoting enhanced de novo lipogene-sis (FASN and SCD1), increased fatty acid uptake (CD36 and FABP4), and reduced PPARα-mediated fatty acid β-oxidation. These coordinated molecular alterations are accompanied by increased intracellular lipid accumula-tion and contribute to the development of MASLD. Conversely, activation of RXR-associated signaling by 9-*cis*-retinoic acid is proposed to attenuate these alterations by suppressing lipogenesis and fatty acid uptake while promoting fatty acid oxidation, thereby reducing hepatic lipid accumulation. Because 9-*cis*-retinoic acid is a pan-RXR agonist capable of activating multiple RXR-dependent signaling pathways, the proposed mechanism should be regarded as a working model that requires receptor-specific mechanistic validation. In the schematic, arrows indicate positive regulation or the direction of change, whereas T-shaped lines indicate negative regu-lation or inhibition.

Figure 8 summarizes the proposed working model derived from the principal findings of the present study. FFA-induced upregulation of miR-34a was associated with reduced RXRα expression, consistent with altered RXR-associated metabolic signaling. Reduced RXRα expression was associated with increased expression of FASN, SCD1, FABP4, and CD36, together with reduced PPARα-associated fatty acid oxidation, resulting in increased hepatic lipid accumulation. In contrast, treatment with 9-*cis*-retinoic acid (9-*cis*-RA) attenuated these molecular alterations and reduced FFA-induced lipid accumulation, consistent with the involvement of RXR-associated signaling in hepatic lipid homeostasis. Figure 8 integrates these findings into a proposed working model of the miR-34a–RXRα regulatory axis in MASLD.

## 3. Discussion

MASLD is a complex metabolic disorder characterized by excessive hepatic lipid accumulation, chronic inflammation, and progressive metabolic dysfunction [1,2]. Despite its increasing global prevalence, effective pharmacological therapies remain limited, highlighting the need to identify novel molecular mechanisms and therapeutic targets [3]. In the present study, we demonstrated that FFA-induced upregulation of miR-34a was associated with reduced RXRα expression and increased hepatic lipid accumulation in steatotic hepatocytes. Furthermore, pharmacological activation of RXR-associated signaling by the pan-RXR agonist 9-*cis*-retinoic acid (9-*cis*-RA) attenuated lipid accumulation and reduced the expression of key lipogenic and fatty acid uptake proteins. Collectively, these findings are consistent with the involvement of the miR-34a–RXRα axis as one regulatory component within a broader miR-34a-regulated nuclear receptor network associated with hepatic lipid homeostasis.

Consistent with previous clinical and experimental studies [8,9,14], miR-34a was markedly upregulated in FFA-induced steatotic hepatocytes and is widely recognized as a stress-responsive microRNA that regulates lipid metabolism, mitochondrial function, apoptosis, and metabolic adaptation [10,11,13]. Previous studies have established SIRT1 and PPARα as major downstream targets of miR-34a, linking elevated miR-34a expression to impaired fatty acid oxidation and hepatic lipid accumulation [26,27,33]. In addition to these established targets, our proteomic analysis demonstrated reduced FOXO3 abundance following miR-34a overexpression, consistent with previous reports identifying FOXO3 as a direct miR-34a target [34]. Together, these findings reinforce the concept that miR-34a coordinately regulates multiple metabolic transcriptional regulators rather than acting through a single downstream effector.

Although RXRα has previously been reported as a direct target of miR-34a, the present study builds upon these previous observations by providing an integrated molecular characterization of the miR-34a–RXRα regulatory relationship. Dual-luciferase reporter assays confirmed direct binding of miR-34a to two conserved binding sites within the RXRα 3′-UTR [20,21]. In contrast, complementary proteomic and transcriptomic analyses consistently identified LXR/RXR signaling as one of the most prominently enriched pathways following miR-34a dysregulation or 9-*cis*-RA treatment. These findings support the interpretation that RXRα functions as one component of a broader miR-34a-regulated nuclear receptor network rather than as the sole mediator of the observed metabolic phenotype. Given the central role of RXRα as an obligate heterodimeric partner of PPARs, LXRs, FXR, and RARs [15,16,17], modulation of RXR-associated signaling may influence multiple interconnected pathways that govern lipid metabolism and metabolic homeostasis [19,35].

The present findings further suggest the potential therapeutic relevance of RXR-associated signaling, as supported by the ability of 9-*cis*-RA to attenuate FFA-induced lipid accumulation and reduce the expression of key regulators of de novo lipogenesis (FASN, SCD1) and fatty acid uptake (FABP4, CD36) [36,37,38,39]. Transcriptomic pathway analysis additionally predicted coordinated modulation of cholesterol transport, bile acid synthesis, and inflammatory signaling, including reduced activity of NF-κB-associated pathways, consistent with previous studies demonstrating the involvement of RXR-associated signaling in regulating hepatic lipid homeostasis and inflammatory responses [40]. Although these pathway predictions require further experimental validation, they are consistent with the established biological functions of RXR-containing nuclear receptor complexes and suggest that RXR-associated signaling may simultaneously regulate metabolic and inflammatory responses in MASLD [18,41,42]. Taken together, our findings support a mechanistic model in which elevated miR-34a contributes to hepatic lipid accumulation through coordinated suppression of multiple metabolic regulators, including SIRT1, PPARα, and RXRα. Rather than functioning through a single downstream target, miR-34a appears to influence multiple interconnected nuclear receptor pathways associated with fatty acid oxidation, lipid synthesis, and metabolic homeostasis [9,27]. This integrated regulatory framework provides a plausible explanation for the widespread transcriptomic and proteomic alterations observed in steatotic hepatocytes.

From a translational perspective, the miR-34a–RXRα axis represents a potential therapeutic target for MASLD. Pharmacological modulation of upstream regulatory pathways may provide broader metabolic benefits than targeting individual downstream enzymes, suggesting potential applications for RXR agonists and next-generation rexinoids [43,44,45]. Nevertheless, several important limitations should be acknowledged.

First, although dual-luciferase assays confirmed direct binding of miR-34a to the RXRα 3′-UTR, genetic rescue experiments restoring miRNA-resistant RXRα expression were not performed. Such experiments will be required to establish whether RXRα is the principal mediator of the observed steatotic phenotype [25]. Second, because 9-*cis*-RA activates both RXR and RAR signaling and RXR forms heterodimers with multiple nuclear receptors [16], the anti-steatotic effects observed in this study cannot be attributed exclusively to RXRα. Receptor-specific loss-of-function approaches, including RXRα knockdown and selective pharmacological modulation, will therefore be necessary to define the contribution of RXRα-dependent signaling. Third, the iTRAQ proteomic analysis was performed as an exploratory, hypothesis-generating study using two biological replicates per group. However, the principal findings were independently validated using dual-luciferase reporter assays and Western blot analyses. Fourth, the present study was conducted primarily in HepG2 cells, which do not fully recapitulate normal hepatocyte physiology [46]. Although the lipid-lowering effects of 9-*cis*-RA were reproduced in primary human hepatocytes, further validation in primary hepatocytes, liver organoids, animal models, and clinical specimens will be required to establish physiological and translational relevance. Finally, complementary biochemical quantification of intracellular triglyceride content, validation of endogenous RXRα protein following miR-34a overexpression, and anti-miR-34a rescue experiments remain important objectives for future investigation.

Recent studies further suggest that retinoic acid (RA) functions as an important immunometabolic regulator within the spleen–liver axis by maintaining splenic dendritic cells, marginal-zone macrophages, and T-cell homeostasis, thereby limiting systemic inflammatory signals that contribute to hepatic inflammation and fibrogenesis [47]. In addition, RA has been reported to directly protect hepatocytes by suppressing RIG-I-dependent TNF-α production and necroptotic signaling [48]. Although these immunological mechanisms were not investigated in the present hepatocyte-based study, they provide a broader biological framework supporting the potential therapeutic role of RA in MASLD and warrant further validation in immune-competent animal models.

Overall, the present study provides an integrated characterization of the miR-34a–RXRα regulatory relationship and supports a model in which elevated miR-34a contributes to hepatic lipid accumulation, at least in part, by suppressing RXR-associated signaling. Pharmacological activation of RXR-associated signaling by 9-*cis*-retinoic acid attenuated steatotic phenotypes in FFA-treated hepatocytes and was associated with coordinated changes in lipid metabolic pathways. Together, these findings provide a framework for future mechanistic studies and translational evaluation of RXR-associated signaling in MASLD. Nevertheless, receptor-specific validation and confirmation in more physiologically representative experimental models will be essential before clinical translation.

## 4. Materials and Methods

### 4.1. Cell Culture and Induction of Hepatic Steatosis

The human hepatocellular carcinoma cell line HepG2 was obtained from the Bioresource Collection and Research Center (BCRC, Hsinchu, Taiwan). HepG2 cells were cultured in Dulbecco’s Modified Eagle Medium (DMEM; Gibco, Grand Island, NY, USA) supplemented with 10% fetal bovine serum (FBS; Gibco) and 1% penicillin–streptomycin, and maintained at 37 °C in a humidified incubator containing 5% CO_2_.

Primary human hepatocytes (HH; Cat. No. 5200, ScienCell Research Laboratories, Carlsbad, CA, USA) were cultured in Hepatocyte Medium (HM; Cat. No. 5201, ScienCell Research Laboratories) according to the manufacturer’s instructions. Cells were seeded onto poly-L-lysine-coated culture plates at the recommended density and maintained at 37 °C in a humidified incubator containing 5% CO_2_. Because primary hepatocytes have limited proliferative capacity in culture, experiments were performed promptly after cell attachment, and the cells were not passaged, in accordance with the manufacturer’s recommendations.

To establish an in vitro steatosis model, free fatty acids (FFAs) were prepared by conjugating oleic acid and palmitic acid (2:1 molar ratio) to fatty acid-free bovine serum albumin (BSA) at an FFA: BSA molar ratio of approximately 6:1. Cells were treated with FFAs at a final concentration of 1 mM for 24 h (corresponding to approximately 0.67 mM oleic acid and 0.33 mM palmitic acid), and matched BSA/vehicle controls were included in all experiments.

For pharmacological activation studies, FFA-treated cells were subsequently incubated with 9-*cis*-retinoic acid (9-*cis*-RA; Sigma-Aldrich, St. Louis, MO, USA) at final concentrations of 1, 5, or 10 μM for 24 h. 9-*cis*-RA was dissolved in DMSO, and the final solvent concentration in the culture medium was maintained below 0.1% (*v*/*v*) and matched across all experimental groups. To minimize isomerization and oxidation, 9-*cis*-RA stock solutions were prepared under reduced light in amber tubes and used immediately after preparation. Cell viability under these treatment conditions had been confirmed previously, ensuring that the concentrations of 9-*cis*-RA used were non-cytotoxic throughout the treatment period.

### 4.2. miR-34a Overexpression and Reporter Plasmid Construction

Transient miR-34a overexpression was achieved using synthetic miR-34a mimics and corresponding negative-control oligonucleotides (Thermo Fisher Scientific, Waltham, MA, USA). Transfections were performed using Lipofectamine™ 3000 (Invitrogen, Carlsbad, CA, USA) according to the manufacturer’s instructions. For stable overexpression, HepG2 cells were transfected with a miR-34a precursor expression construct and selected with G418 to establish stable cell lines.

To evaluate direct miRNA–target interactions, fragments of the human PPARα, SIRT1, and RXRα 3′-untranslated regions (3′-UTRs) containing the predicted miR-34a binding sites were cloned into the pMIR-REPORT vector (Applied Biosystems, Foster City, CA, USA). All constructs were verified by DNA sequencing before use in dual-luciferase reporter assays.

### 4.3. Dual-Luciferase Reporter Assay

HepG2 cells were seeded in 24-well plates and co-transfected with wild-type or mutant pMIR-REPORT reporter constructs containing the PPARα, SIRT1, and RXRα 3′-untranslated regions (3′-UTRs), together with synthetic miR-34a mimics or negative-control oligonucleotides, using Lipofectamine™ 3000 (Invitrogen) according to the manufacturer’s instructions. A Renilla luciferase plasmid was co-transfected as an internal control for normalization. After 48 h, firefly and Renilla luciferase activities were measured using the Dual-Glo^®^ Luciferase Assay System (Promega, Madison, WI, USA). Firefly luciferase activity was normalized to Renilla luciferase activity, and the results were expressed as normalized relative luciferase activity [49].

### 4.4. Protein Extraction and Western Blot Analysis

Total cellular proteins were extracted using RIPA lysis buffer supplemented with protease and phosphatase inhibitor cocktails. Protein concentrations were determined using the Bradford protein assay. Equal amounts of total protein (100 μg) were separated by SDS–PAGE and electrotransferred onto polyvinylidene fluoride (PVDF) membranes. The membranes were blocked with 5% non-fat milk in Tris-buffered saline containing 0.1% Tween-20 (TBST) for 1 h at room temperature and then incubated overnight at 4 °C with primary antibodies against RXRα (Cat. No. A15242, ABclonal, Wuhan, China), SIRT1 (Cat. No. A11267, ABclonal), α-tubulin (Cat. No. AC012, ABclonal), CD36 (Cat. No. A1470, ABclonal), FABP4 (Cat. No. A0232, ABclonal), SCD1 (Cat. No. A26246, ABclonal), PPARα (Cat. No. A18252, ABclonal), FASN (Cat. No. A21182, ABclonal), and GAPDH (Cat. No. AC033, ABclonal). Membranes were subsequently incubated with horseradish peroxidase (HRP)-conjugated anti-rabbit IgG (Cat. No. 7074, Cell Signaling Technology, Danvers, MA, USA) or anti-mouse IgG (Cat. No. 7076, Cell Signaling Technology) secondary antibodies for 1 h at room temperature. Immunoreactive bands were detected using an enhanced chemiluminescence (ECL) detection system (GE Healthcare, Chicago, IL, USA). Band intensities were quantified using ImageJ software (version 1.54, National Institutes of Health, Bethesda, MD, USA), normalized to the corresponding loading control (α-tubulin or GAPDH), and expressed as fold changes relative to the corresponding control group.

### 4.5. Oil Red O Staining and Lipid Quantification

Intracellular lipid accumulation was assessed by Oil Red O staining as previously described with minor modifications [50]. Following the indicated treatments, cells were fixed with 10% neutral-buffered formalin and stained with a freshly prepared, filtered Oil Red O working solution. Representative images of intracellular lipid droplets were acquired using a light microscope. For quantitative analysis, the retained Oil Red O dye was eluted with isopropanol, and absorbance was measured at 510 nm using a microplate reader. The absorbance values were normalized to total cellular protein concentration to normalize for variations in cell number.

### 4.6. iTRAQ-Based Proteomic Analysis

Total cellular proteins from miR-34a-overexpressing and control HepG2 cells were digested with trypsin and labeled using an 8-plex iTRAQ reagent kit (AB Sciex, Framingham, MA, USA). Labeled peptides were fractionated by high-performance liquid chromatography (HPLC) and analyzed by liquid chromatography–tandem mass spectrometry (LC–MS/MS). Protein identification and quantification were performed using Proteome Discoverer software (version 1.4, Thermo Fisher Scientific, Waltham, MA, USA).

Differentially expressed proteins (DEPs) were screened using abundance ratio thresholds of ≥1.20 or ≤0.83, which are widely used in iTRAQ-based proteomic studies to identify biologically relevant expression changes. Because only two biological replicates were included per group, these fold-change thresholds were applied as exploratory screening criteria rather than formal measures of statistical significance. Candidate DEPs were subsequently analyzed using Ingenuity Pathway Analysis (IPA; Qiagen, Hilden, Germany), and key findings were independently validated by Western blot analysis.

### 4.7. RNA Sequencing and Bioinformatic Analysis

Differential gene expression analysis was performed using DESeq2 with significance thresholds of an adjusted *p* < 0.05 and |log_2_ fold change| > 1. Canonical pathway and upstream regulator analyses were conducted using Ingenuity Pathway Analysis (IPA; Qiagen, Hilden, Germany). Putative miR-34a target genes were identified using TargetScan, with miRNA annotation referenced from miRBase.

### 4.8. Statistical Analysis

Unless otherwise specified, all experiments were performed using at least three independent biological replicates, and data are presented as the mean ± standard deviation (SD). Statistical analyses were performed using GraphPad Prism version 6.0 (GraphPad Software, San Diego, CA, USA). Comparisons between two groups were analyzed using an unpaired two-tailed Student’s *t*-test. In contrast, comparisons among multiple groups were performed using one-way analysis of variance (ANOVA) followed by Tukey’s multiple-comparison post hoc test. For RNA sequencing, differential gene expression analysis was performed using DESeq2 with Benjamini–Hochberg adjustment for multiple testing. A two-sided *p* < 0.05 was considered statistically significant.

## 5. Conclusions

In summary, we identified RXRα as a direct target of miR-34a and provided an integrated proteomic and transcriptomic characterization of the miR-34a–RXRα regulatory axis. Our findings demonstrate that elevated miR-34a expression is associated with reduced RXRα expression and increased hepatic lipid accumulation, supporting the concept that RXRα functions as one regulatory component within a broader miR-34a-regulated nuclear receptor network. Pharmacological activation of RXR-associated signaling with 9-*cis*-retinoic acid was associated with attenuation of FFA-induced lipid accumulation, along with coordinated changes in pathways involved in lipid uptake, de novo lipogenesis, and metabolic homeostasis. Collectively, these findings provide mechanistic insight into the role of the miR-34a–RXRα axis in hepatic lipid metabolism and support RXR-associated signaling as a promising therapeutic strategy for MASLD. Nevertheless, receptor-specific validation and confirmation in more physiologically representative experimental models will be required before the therapeutic potential of this regulatory axis can be translated toward clinical application.

## Data Availability

The datasets generated and/or analyzed during the current study are available from the corresponding author upon reasonable request. The RNA sequencing (RNA-seq) dataset has been deposited in the NCBI Gene Expression Omnibus (GEO) database under accession number GSE337589 and is publicly available.

## Supplementary Materials

The following supporting information can be downloaded at: https://www.mdpi.com/article/10.3390/ijms27156609/s1.

## Abbreviations

The following abbreviations are used in this manuscript:

MASLD	metabolic dysfunction-associated steatotic liver disease
MAFLD	metabolic dysfunction–associated fatty liver disease
NAFLD	nonalcoholic fatty liver disease
MASH	metabolic dysfunction-associated steatohepatitis
miR-34a	microRNA-34a
RXRα	retinoid X receptor alpha
9-*cis*-RA	9-*cis*-retinoic acid
FFA	free fatty acid
LXR	liver X receptor
PPARα	peroxisome proliferator-activated receptor alpha
iTRAQ	isobaric tags for relative and absolute quantitation
IPA	Ingenuity Pathway Analysis
DEG	differentially expressed gene
3′-UTR	3′ untranslated region
