# Supplementary material for: MicroRNA-34a Promotes Hepatic Lipid Accumulation Through RXRα Suppression and Is Reversed by 9-cis-Retinoic Acid in Steatotic Hepatocytes
Source: Int J Mol Sci. 2026 Jul 24;27(15):6609. doi: 10.3390/ijms27156609 (PMC13465280; doi:10.3390/ijms27156609)
Supplement: Supplementary file 1 [file ijms-27-06609-s001.zip › ijms-4415738-supplementary.pdf]

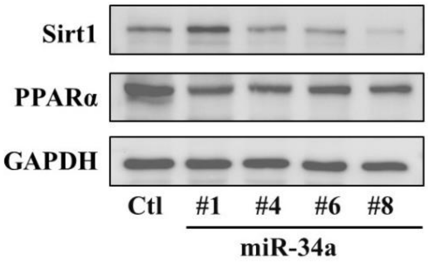

Figure S1. Stable overexpression of miR-34a progressively suppresses SIRT1 and PPARα protein levels in HepG2 cells. Representative Western blots demonstrate dose-dependent reduction in SIRT1 and PPARα protein in HepG2 cells stably expressing miR-34a (clones #1, #4, #6, and #8) relative to the empty-vector control (Ctl). GAPDH served as the loading control. The graded suppression across independent clones supports the on-target silencing of SIRT1 and PPARα by miR-34a.

Table S1. iTRAQ-based quantitative proteomic profiling of miR-34a-overexpressing HepG2 cells. The complete proteomic dataset (Supplementary_Table_S1-iTRAQ.xlsx) contains 6,907 identified proteins, of which 6,623 were quantitatively analyzed in miR-34a-overexpressing and control HepG2 cells. For each protein, the dataset includes the UniProt accession number, protein description, sequence coverage (%), peptide count, peptide-spectrum matches (PSMs), unique peptide count, amino acid length, molecular weight (kDa), theoretical isoelectric point (pI), Sequest HT score, gene symbol, abundance ratio (Sample/Control), adjusted p-value, and grouped abundance values for each experimental condition. Based on abundance ratio thresholds of ≥1.2 and ≤0.83, 2,508 proteins were classified as upregulated and 1,610 as downregulated in miR-34a-overexpressing cells. FOXO3 and FOXO-associated signaling components are included among the quantified proteins, with FOXO3 exhibiting reduced abundance in miR-34a-overexpressing cells.

See in supplementary excel files Table S1.

Table S2. RNA sequencing differential expression dataset of FFA-treated HepG2 cells following RXR-associated signaling. The complete transcriptomic dataset (Supplementary Table S2-RNAseq.xlsx) contains 60,623 Ensembl gene entries annotated with Ensembl gene identifiers and official gene symbols. Differential gene expression is presented as $\log_2$ fold-change values across multiple pairwise comparisons, including FFA-treated HepG2 cells co-treated with 9-cis-retinoic acid (9-cis-RA) relative to untreated HepG2 cells or FFA-treated controls. The dataset also includes the comparison between FFA-treated and untreated HepG2 cells. For the primary comparison of 9-cis-RA-treated versus FFA-treated cells, 18,836 genes contained valid differential expression values, of which 551 were upregulated, and 826 were downregulated using a cutoff of $|\log_2$ fold change$| > 1$.

See in supplementary excel files Table S2.
